# The Crusade against Tuberculosis

**Published:** 1901-08

**Authors:** 


### EDITORIAL NOTES AND COMMENTS.

The Business office of THE JOURNAL-RECORD is No. 64 Marietta Street.

The Editorial office is 318-319-320 The Prudential.

Address all Business communications to Dr. M. B. Hutchins, Mgr.

Make remittances payable to THE ATLANTA JOURNAL-RECORD OF MEDICINE.

On matters pertaining to the Editorial and Original Communications address Dr. Bernard Wolff, Atlanta.

Reprints of original articles will be furnished at cost price. Requests for the same should always be made *on the manuscript*.

We will present, post-paid, on request, to each contributor of an original article, twenty (20) marked copies of THE JOURNAL-RECORD containing such article.

### THE CRUSADE AGAINST TUBERCULOSIS.

APART from the frankly and demonstrably communicable diseases there is no one which of late has been more marked as an object of attention from the point of view of repression as tuberculosis. The fact that this disease causes the death of one-third of the human family would appear to be motive enough for waging ceaseless war against it, but the conflict is stimulated by the fact that tuberculosis is relatively preventable. When a disease falls in this category, a death from it seems an unnecessary sacrifice; and sacrafices have no place in the scheme of modern economics. To prevent the spread of the disease, many suggestions have been offered of more or less feasibility. The question of legislative enactment aimed at arresting the promulgation of the disease without violent infringement upon individual right and privilege engages the attention as a matter of prime importance. In order for the enforcement of such preventive legislation to be effective it must be backed up by a strongly favorable public sentiment. Lacking this it will inevitably become rapidly obsolete. The general opinion prevails that the time is not ripe for such legislation. Every one recognizes the wisdom of it, but they realize at the same time that it is as yet inopportune. The public must be educated up to an intelligent knowledge of the disease largely by the dissemination of literature and instruction by the medical profession. It goes without saying that it will require much time. The public is swayed by sentiment rather than reason, and until fear, the most primitive and strongest of the instincts, predominates, the task will be incomplete and the practical solution of the problem unfinished. This applies of course to the passage of drastic measures and does not include such restrictive measures as affect the transmission of tuberculosis from animals to man, and ordinary sanitary precautions relating to the consumptive person. Every one will admit that these are not only necessary, but eminently reasonable. What will be the effect of the various general conferences on the subject of tuberculosis which have taken place and are to take place is not yet apparent, but it is to be hoped that some practical results may come from them, and that their deliberations will not go to add to the dusty heaps of what Carlyle calls shot-rubbish and Torpedoism. Whatever their outcome be, it is a sign of promise, and one that indicates that the profession is putting its shoulder to the wheel for a revolution in the right direction.

